# *openFrame*: A modular, sustainable, open microscopy platform with single‐shot, dual‐axis optical autofocus module providing high precision and long range of operation

**DOI:** 10.1111/jmi.13219

**Published:** 2023-09-27

**Authors:** J. Lightley, S. Kumar, M. Q. Lim, E. Garcia, F. Görlitz, Y. Alexandrov, T. Parrado, C. Hollick, E. Steele, K. Roßmann, J. Graham, J. Broichhagen, I. A. McNeish, C. A. Roufosse, M. A. A. Neil, C. Dunsby, P. M. W. French

**Affiliations:** ^1^ Photonics Group, Physics Department Imperial College London London UK; ^2^ Francis Crick Institute London UK; ^3^ Department of Surgery and Cancer Imperial College London London UK; ^4^ Cairn Research Ltd Faversham Kent England; ^5^ Leibniz‐Forschungsinstitut für Molekulare Pharmakologie Berlin Germany; ^6^ Department of Inflammation and Immunology Imperial College London London UK

**Keywords:** autofocus, fluorescence microscopy, open‐source microscopy, superresolution, sustainability, widening participation

## Abstract

‘*openFrame*’ is a modular, low‐cost, open‐hardware microscopy platform that can be configured or adapted to most light microscopy techniques and is easily upgradeable or expandable to multiple modalities. The ability to freely mix and interchange both open‐source and proprietary hardware components or software enables low‐cost, yet research‐grade instruments to be assembled and maintained. It also enables rapid prototyping of advanced or novel microscope systems. For long‐term time‐lapse image data acquisition, slide‐scanning or high content analysis, we have developed a novel optical autofocus incorporating orthogonal cylindrical optics to provide robust single‐shot closed‐loop focus lock, which we have demonstrated to accommodate defocus up to ±37 μm with <200 nm accuracy, and a two‐step autofocus mode which we have shown can operate with defocus up to ±68 μm. We have used this to implement automated single molecule localisation microscopy (SMLM) in a relatively low‐cost *openFrame*‐based instrument using multimode diode lasers for excitation and cooled CMOS cameras.

## LAY DESCRIPTION

We present a modular, low‐cost, microscopy platform called ‘*openFrame*’ that can be configured or adapted to most light microscopy techniques and is easily upgradeable. *openFrame*‐based microscopes are designed to be used with open‐source software tools and low‐cost/self‐built hardware components, such that cost‐effective instruments can be assembled and maintained locally by skilled users instead of requiring visits from service engineers. The core *openFrame* hardware components can be fabricated using freely shared CAD files or they can be purchased by users to implement a wide range of instruments, which can utilise a mixture of open and proprietary hardware components as desired. We intend this platform to widen access to basic and advanced microscopy techniques, including in lower‐resource settings. We also expect it to be useful to researchers prototyping new microscopes, since it allows a core instrument to be rapidly assembled and then easily modified with minimal constraints, for example, from proprietary software or legacy design choices. The flexibility to reconfigure *openFrame*‐based microscopes and interchange/recycle components between different instruments reduces obsolescence. The *openFrame*‐based microscope presented here includes a novel open‐source optical autofocus unit (*openAF*) that may be useful for long‐term time‐lapse imaging, and in slide‐scanning or automated multiwell plate microscopes. We demonstrate the use of an *openFrame*‐based instrument for super‐resolved microscopy using single molecule localisation microscopy (SMLM) utilising low‐cost cooled CMOS cameras and multimode diode lasers for high power excitation.

## 1 INTRODUCTION

Optical microscopy is a ubiquitous tool in research for a diverse range of applications, and the development of automated instrumentation for high content analysis provides opportunities to accelerate discovery. Recent decades have seen tremendous advances in imaging capabilities but access to many advanced microscopy modalities can be prohibited by their cost to purchase and/or lack of support for lower‐resource research settings. This is being addressed by open‐source approaches – predominantly through open‐source software tools, but increasingly complemented by open microscopy hardware, which is extensively reviewed in Ref. (1). Open hardware and software can also provide flexibility for instrument developers when designing bespoke optical systems. Motivated both to reduce the cost and time required to develop new instruments and to widen access to advanced microscopy techniques, we have developed a low‐cost, modular, open‐source, generic microscopy platform based around a modular microscope frame (‘*openFrame*’) on which we mount cost‐effective commercial or self‐built components, which we typically control using *μManager* ^2^ software. Routine image analysis is undertaken using *ImageJ*.^3^ The recent availability of sensitive cooled CMOS cameras combined with LED or multimode diode laser sources enables fluorescence or quantitative phase microscopes^4^ to be assembled using *openFrame* at relatively low cost. In our laboratory, we are exploring the use of *openFrame*‐based microscopes to implement cost‐effective open‐source instruments for specific research applications such as single molecule localisation microscopy (SMLM), histopathology (integrating bright‐field and immunofluorescence microscopy) and automated imaging of samples arrayed in multiwell plates for high content analysis (HCA).

In practice, many light microscopy modalities can be compromised by thermal and other fluctuations that can lead to drift of components over time such that the desired field of view (FOV) becomes defocused. This can be a particular problem for experiments with long image data acquisition times, including SMLM, and is an essential consideration for automated slide‐scanning or multiwell plate imaging where the sample may not be in the same horizontal plane for every FOV during the image data acquisition. We previously addressed this challenge by developing a long‐range (dual‐axis) optical autofocus module that provided high precision (within the depth of field of high numerical aperture objective lenses) over a wide range of initial defocus (approximately ±100 μm) and utilised machine learning to determine the sign and quantify the degree of defocus prior to correction in a two‐step process.^5^ This autofocus worked well – requiring no significant adjustment over >12 months – but the set up required the acquisition of training data over ∼2 weeks to be able to build a CNN to account for long‐term system drift, and this implementation of a dual‐axis autofocus did not operate in single‐shot/closed‐loop mode. Here we present a refinement of this approach that does not require machine learning and provides robust performance over short and long ranges with single‐shot/closed‐loop capability.

## 2 openFrame

**FIGURE 1:**
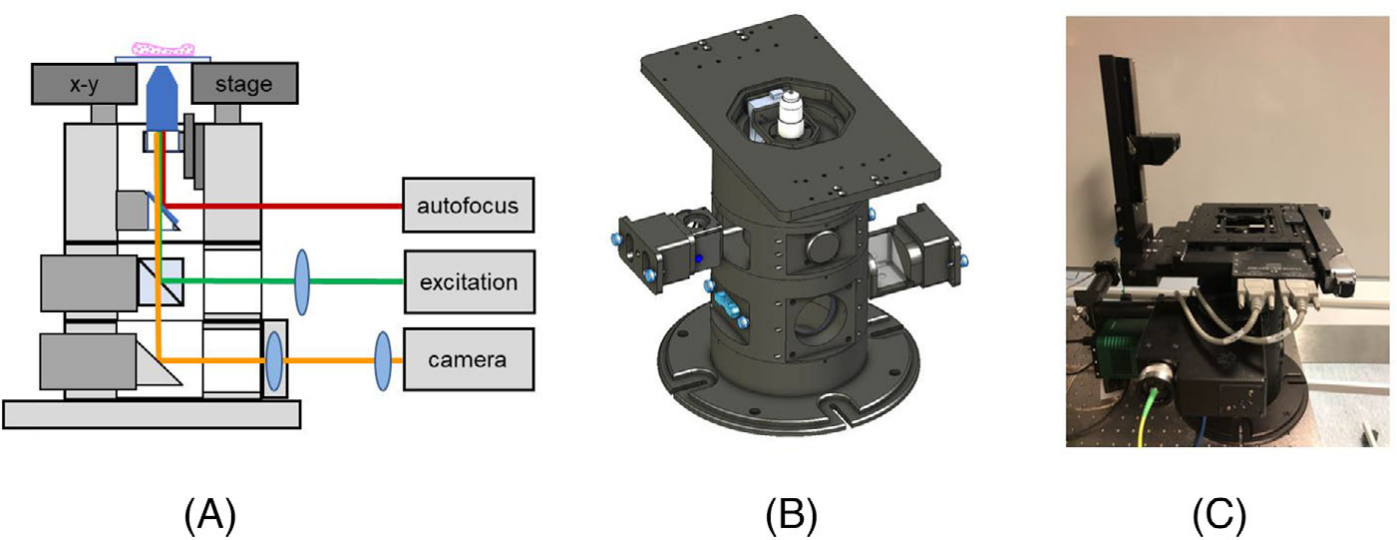
(A) schematic of *openFrame*‐based fluorescence microscope, (B) render of CAD of core *openFrame* components and (C) photograph of *openFrame‐*based microscope configured for automated *easySTORM*.^5^

As illustrated in Figure 1, the *openFrame* concept is based on a microscope stand with a cylindrical layered design centred around the main optical axis. Layers incorporating elements such as dichroic beamsplitters, mirrors and lenses can be stacked to create the desired microscope functionality, which can encompass multiple illumination/excitation and imaging paths as required. The layers are machined from aluminium and are clamped together to realise a rigid instrument of comparable mass and stability as commercial research‐grade fluorescence microscope frames. Additional components, including cameras, light sources, optomechanical cage assemblies or breadboard‐based subsystems, can be securely bolted to the *openFrame* stand to implement different microscopy or other instrumentation modalities. As indicated in the Supplementary Information, the *openFrame* GitHub repository at https://github.com/ImperialCollegeLondon/openFrame is where CAD files for these cylindrical layers and other core *openFrame* components are freely shared^6^ under the permissive version of the CERN Open Hardware License Version 2 (https://ohwr.org/cern_ohl_p_v2.pdf), along with related resources for those users who wish to manufacture them to assemble their own microscopes or companies who may wish to include *openFrame* components in their commercial instruments or attach their commercial modules to *openFrame* microscopes. Alternatively, *openFrame* components can be purchased (e.g., from Cairn Research Ltd) and used without restriction.

*openFrame* represents a cost‐effective compromise between open hardware self‐built from generic optomechanical components and commercial microscopes. Because the *openFrame* components are specifically designed for microscopy, a functional instrument can be rapidly assembled with little specialist knowledge, and at a lower cost than building from scratch with generic optomechanical components. The open, modular nature of *openFrame* makes it easier to maintain and upgrade/repurpose than commercial microscopes. *openFrame* microscopes can be highly cost‐effective because it is only necessary to purchase (or fabricate) the components needed for a particular experiment – or to use existing standard microscope components, including microscope stages, light sources, optical components and detectors – but the instrument can be later extended to realise different or more complex multimodal instruments. It is straightforward to ‘mix and match’ self‐built and commercially available components, and we anticipate that a range of open‐hardware projects will provide lower cost alternatives to many commercial products where users are prepared to assemble their own subsystems. Decreasing reliance on proprietary instruments and software makes it easier to maintain and/or upgrade instruments, which can be important for users in lower‐resource settings and/or where there is limited access to technical support. In highly resourced laboratories, *openFrame* can accelerate prototyping of new instruments since its modular design is fully open and the hardware can be rapidly reconfigured for a new modality.

The *openFrame* concept was conceived following our development of *easySTORM* ^7^ – a low‐cost implementation of direct STORM (dSTORM)^8^ or ground state depletion microscopy followed by individual molecule return (GSDIM)^9^ utilising multimode diode lasers and multimode optical fibres – and our subsequent efforts to make SMLM accessible to scientists in lower‐resource settings. Noting that SMLM can take advantage of low‐cost CMOS cameras,^10^, ^11^ we observed that a commercial microscope frame would become the most expensive component of a research‐grade SMLM microscope (or most fluorescence microscopes) and sought to reduce this cost to enable our open‐source instruments to be used in lower‐resource settings, where it may not be possible to purchase or to support commercial research‐grade fluorescence microscopes. Figure 1 presents a schematic of the basic components required to realise an epifluorescence microscope and the components that can be used to assemble a low‐cost *easySTORM* microscope are shared in Supplementary Information. In our laboratory, we are developing automated multiwell plate SMLM instruments and therefore mount a motorised *x*–*y* translation microscope stage on the *openFrame* stand and use a long‐travel piezoelectric actuator for *z*‐translation of the microscope objective lens – including focus lock functionality using the optical autofocus system described below. We note that the *x*–*y* translation stage and *z*‐drive we have used are commercial products that we have mounted on the *openFrame* microscope, but in principle any *x*–*y* translation stage or *z*‐drive can be used as long as they fulfil the user requirements. However, a simpler and cheaper implementation can be realised using a manual *x*–*y*–*z* stage to hold the sample. Although our microscopes are primarily controlled using *μManager* ^2^ to take advantage of the rich legacy of hardware plug‐ins, in principle, other software tools, for example, Python‐based microscopy control software,^12^, ^13^, ^14^, ^15^ could be used to control *openFrame*‐based hardware, subject to the required hardware drivers or device adapters being available or written as needed, along with a suitable user interface in the instrument control software.

The open microscopy hardware field is evolving rapidly^1^, ^16^, ^17^, ^18^, ^19^ and the availability of low‐cost excitation sources based on LED or diode lasers is complemented by developments in low‐cost CMOS cameras. For fluorescence microscopy, including *easySTORM*, we are using two newly available cooled backside‐illuminated CMOS cameras based on circuitry originally developed for the amateur astronomy community and redesigned for the life sciences. These are the CellCam Centro and the CellCam Kikker (Cairn Research Ltd). The Centro is fan‐cooled and is based on the Sony IMX 183 sensor (>80% quantum efficiency, 2.4 μm pixel size, 13.2 × 8.8 mm) while the CellCam Kikker is thermoelectrically cooled and is based on the Sony IMX 492 sensor (90% quantum efficiency, 2.315 μm or 4.63 μm pixel size, 13 × 19 mm). For many practical applications of fluorescence microscopy, we believe that these can be acceptable replacements for scientific CCD or sCMOS cameras. Figure S2 shows a direct comparison of the performance for STORM of the CellCam Kikker, the CellCam Centro and an established sCMOS camera.

## 3 OPTICAL AUTOFOCUS METHODOLOGY

With low‐cost excitation sources, cameras and microscope stands, it is possible to implement basic fluorescence microscopy, which can be useful for many applications. However, for extended image data acquisition, for example, for SMLM or time‐lapse microscopy, or for automated multiwell plate microscopy or slide‐scanning, an autofocus system is required to compensate for thermal or mechanical drift of alignment of optical system components, and to refocus after changing field of view, for example, when scanning a multiwell plate. Autofocus functionality can be most readily achieved using software‐based autofocus techniques that utilise the images normally acquired by the microscope.^20^ Typically, this involves acquiring a set of bright‐field or fluorescence images over a range of defocus and analysing them to select and/or iterate towards the best focus condition. This generally requires the acquisition of new image data at each new FOV, in addition to the image data acquired for intended experiment, which can increase phototoxicity and slow the imaging throughput. The speed of software‐based autofocus approaches typically scales with the axial range over which the autofocus is implemented – which typically scales with the depth of field of the microscope. Such software‐based autofocus may be too slow for applications such as slide‐scanning and high content analysis, are not suitable for samples of extended depth, and may not operate over sufficient range of defocus to accommodate moving between distant fields of view, for example, on a multiwell plate.

A variety of hardware‐based autofocus or ‘optical autofocus’ approaches have been reported that entail modifying the microscope hardware. These typically track and facilitate compensation of the relative axial motion of the coverslip/sample interface with respect to the microscope objective lens. Defocus may be measured through auxiliary imaging of features in the sample^21^ or of fiduciary markers (such as subresolution‐sized beads)^22^, ^23^ or it can be measured using an auxiliary optical system, typically focussing a laser beam onto the coverslip/sample interface and monitoring the back‐reflected light using an auxiliary detector. The use of low power near‐infrared radiation for this can significantly reduce the phototoxic light dose on the sample compared to software‐based techniques using the primary data acquisition camera.

Defocus can be quantified using a metric calculated from measurement of this back‐reflected laser beam such as its power or displacement^24^, ^25^ or size^5^ of image. Such calculated metrics may not distinguish whether the defocus is positive or negative. This may be addressed, for example, by implementing a multistep approach^5^ at the cost of speed. Alternatively, the focussing of the back‐reflected laser beam at the autofocus camera can be offset such that any drift of the sample relative to the microscope objective does not cause the reflected laser beam to pass through focus (minimum spot size) on the autofocus camera,^26^ but this can decrease the operating range of the optical autofocus system.

The precision and operating range of an optical autofocus based on back‐reflections of a laser beam from the microscope coverslip can be adjusted by controlling the diameter of this laser beam in the back aperture of the microscope objective lens – and therefore the autofocus confocal parameter. Normally this entails a compromise between precision and range of the optical autofocus system. We recently demonstrated^5^ that this compromise could be relaxed by using a slit to shape the autofocus laser beam such that it exhibited a different confocal parameter in the planes parallel and perpendicular to the slit. Thus, it was possible to make parallel measurements of defocus with (i) higher precision over a shorter range for faster focussing axis and (ii) lower precision over a longer range for the slower focussing axis. A two‐step algorithm was used to bring the microscope back to focus from anywhere in range of the slower focussing autofocus axis. This enabled us to achieve an accuracy better than the depth of field of the objective lens (∼600 nm) over an operating range of approximately ±100 μm. We further used a convolutional neural network (CNN) to determine the defocus by comparing the autofocus camera image to training data of autofocus camera *z*‐stacks previously recorded when scanning a coverslip through the focal plane of the microscope objective lens. This CNN‐based readout was sufficiently sensitive to the variation of the autofocus camera images that it could also determine the sign of defocus. However, we found that, because the optical system of the autofocus itself was subject to drift, it would only work well with training data acquired within a few hours of the experiment. We overcame this by collecting separate training data over multiple days and forming a composite training data set that encompassed the common perturbations in the alignment of the autofocus system (and therefore the images recorded on the autofocus camera).

Using this composite training data, the CNN‐based optical autofocus module has functioned stably for >12 months. However, it was a significant effort to acquire the composite training data in order to set up the system and it will be necessary to acquire new training data if the microscope objective lens is changed. Also, the two‐step algorithm to correct defocus is not suitable for rapid closed‐loop operation. We therefore decided to develop a new optical autofocus that retains the capability for high precision and long operating range but does not require machine learning and the associated acquisition of training data. It also senses the sign of defocus without compromising the operating range and so can operate in closed‐loop single‐shot mode for real‐time focus control.

**FIGURE 2:**
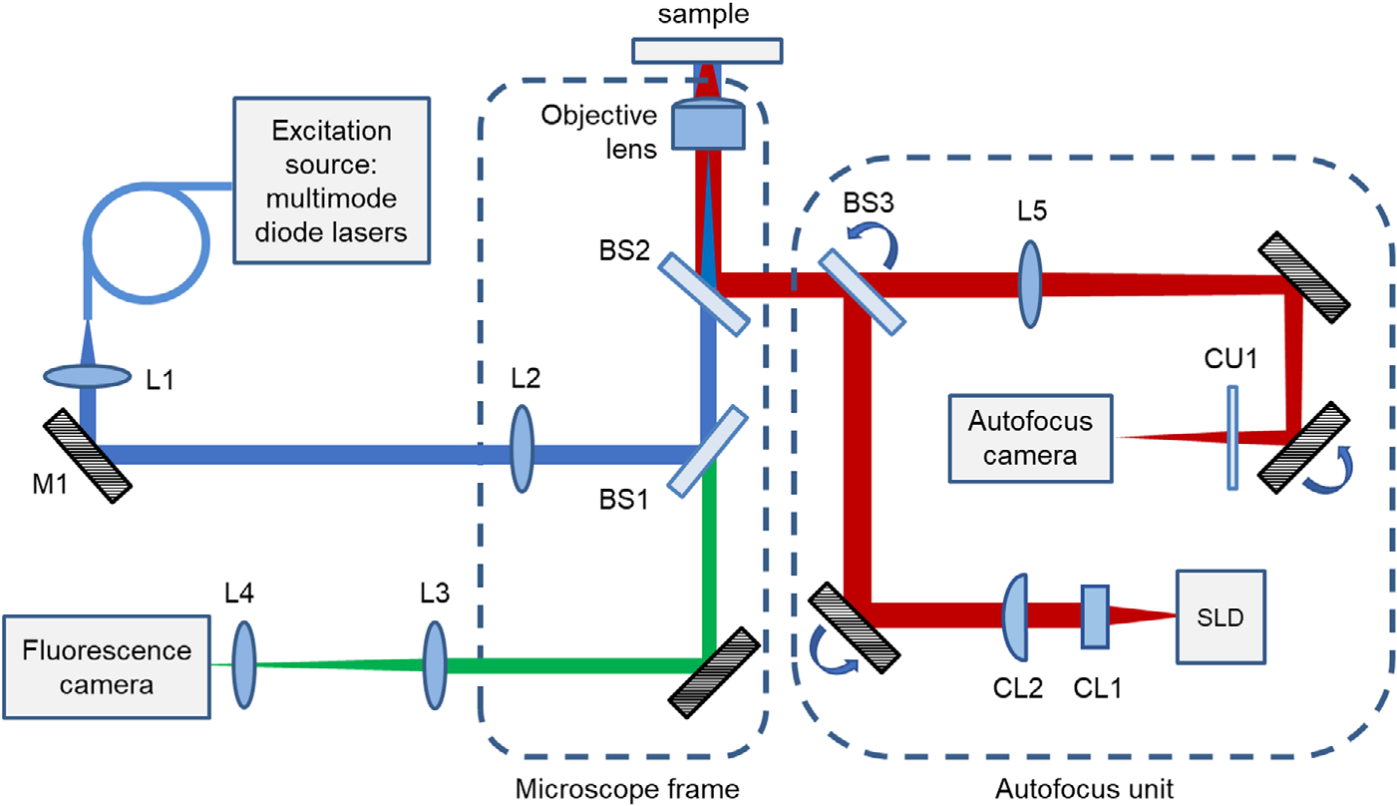
Implementation of the dual‐axis cylindrical lens autofocus on an *openFrame* microscope. A superluminescent laser diode (Superlum S930‐B‐I‐10) was coupled into a single mode fibre with a 0.1 NA and the output of the fibre was used as the source for the autofocus. The beam was collimated in one of the orthogonal axes with a 6.4 mm focal length cylindrical lens (CL1, Thorlabs LJ1227L1‐B) and in the other direction by a 20.0 mm focal length cylindrical lens (CL2, Thorlabs LJ1960L1‐B) to produce an elliptical beam profile. The beam was reflected first by a 50:50 beamsplitter, BS3 (Edmund Optics 47‐026), and then reflected by an 800 nm short pass filter, BS2 (Edmund Optics 69–220). The elliptical beam was then focused onto the sample by an Olympus 100×, 1.4 NA objective lens (Olympus UplanSApo 100×1.40 oil) used with Type F immersion oil and reflected at the refractive index change between the coverslip and the sample's media. The reflected signal then propagated back through the system and was transmitted by the 50:50 beamsplitter, BS3, before being imaged by an auxiliary autofocus camera, (FLIR Chameleon3 CM3‐U3‐31S4M‐CS) by a 200 mm focal length lens L5 (Thorlabs AC254‐200‐B). A longpass clean‐up filter CU1 (COMAR 780 GY 25) was used to limit the light detected by the autofocus camera to the spectral range of the autofocus SLD. The fluorescence excitation radiation is delivered by multimode optical fibre and collimated by a 60 mm lens L1 (Thorlabs AC254‐060‐A) and directed to the sample via beam‐steering mirror M1 and the primary fluorescence microscope dichroic beamsplitter BS1 (Chroma ZT405_465_625_825rpc‐UF2). Lens L2 (Edmund optics 49–364) focuses the excitation to the back aperture of the oil immersion objective lens.

Figure 2 shows the configuration of this new optical autofocus system (designated ‘*openAF*’), which was implemented on an *openFrame* microscope configured for *easySTORM* ^6^, ^7^ using a 100× objective lens of 1.4 NA (Olympus UplanSApo 100× 1.40 oil). The key difference to our previously published optical configuration^5^ is that the autofocus laser beam is collimated after emerging from a single mode optical fibre by a pair of cylindrical lenses of different focal length orientated at 90° to each other to produce the desired elliptical collimated beam (instead of using a spherical collimating lens and a rectangular slit). By using cylindrical lenses with different focal lengths, we can adjust the autofocus confocal parameters of the orthogonal directions independently and thus the precision and operating range of the autofocus. Considering the trade‐off between precision and range of defocus measurement, the choice of focal lengths for the cylindrical lenses was made from stock lenses to keep the precision of defocus measurement within the depth of field of the objective lens. One of the cylindrical lenses is set slightly away from the collimation position to axially offset the foci of the two cylindrical lenses at the coverslip. This breaks the symmetry about the focal plane, which enables the direction of defocus to be determined from a single image since the peaks of the defocus metrics are shifted relative to each other. For readouts of defocus, we bin the autofocus camera images in the directions parallel to the two cylindrical lens axes to generate respective intensity projections.

The defocus can be determined from a single autofocus camera image by previously recording a single *z*‐stack of autofocus camera images as the sample coverslip is translated axially through focus and calculating the FWHM of the Fourier transform power spectrum of each projection at each *z*‐position (defocus value) to create a look‐up table of this metric as a function of defocus. Figure S2 illustrates this process. We chose to use this image metric to quantify defocus as we found it to be less sensitive to background noise, for example, from spurious back‐reflections of autofocus laser light from other components in the optical system, compared to the FWHM or standard deviation of the autofocus camera intensity projections. In general, we avoid image metrics based on displacement of the back‐reflected autofocus laser beam because these can be more sensitive to changes in the optical alignment. Before calculating the FWHM of the Fourier transform power spectrum of each projection, it is necessary to address the unwanted background signal arising from back‐reflections from other components in the optical system. For this, we calculate an average of two background images (previously recorded at a significant distance above and below focus) that we then subtract from subsequently acquired autofocus camera images.

**FIGURE 3:**
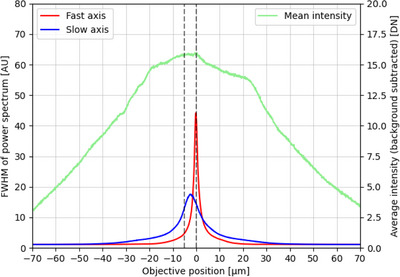
Plots of defocus metrics against axial position (*z*): the FWHM of the Fourier transform power spectrum of the two projections of average intensity along the horizontal and vertical axes of the sensor of the FLIR Chameleon3 CMOS camera. The red and blue peaks represent the autofocus metrics for the short‐range confocal parameter and long‐range confocal parameter respectively. The green plot shows the average autofocus camera pixel intensity in the image after background subtraction. As the 20 mm focal length lens has been positioned slightly further away than its focal length from the fibre, it focuses onto the sample 2 μm axially above the blue peak. This enables closed‐loop single‐shot autofocus. The vertical dashed lines indicate the defocus positions at which the data shown in Figure S2 was acquired.

Figure 3 shows a plot of the two (long‐ and short‐range confocal parameter) image defocus metrics as a function of axial displacement of objective lens. The red curve shows the metric for the camera projections in the plane of the larger collimated beam diameter (shorter autofocus confocal parameter) and the blue curve shows the autofocus camera projections corresponding to the longer autofocus confocal parameter. The average intensity over the whole autofocus camera image after background subtraction is shown in the green curve to give an indication of total signal received as a function of defocus.

For our previous autofocus implementation that used a CNN to process the autofocus camera images, the contributions from any unwanted back‐reflections off components in the autofocus laser path were included in the training image data and did not appear to compromise the system performance. Since this new optical autofocus system is intended to operate without the need for CNN‐based analysis and the requisite training image data, these unwanted back‐reflections can impact the performance. This is because the high spatial and temporal coherence of the single spatial mode autofocus laser beam leads to interference patterns between the main signal reflected from the sample coverslip and spurious reflections from other surfaces in the optical system. These interference patterns can cause spatial modulation of the image recorded on the autofocus camera that changes as the distance from the objective lens to coverslip is varied, for example, due to drift or during an axial scan. Because this unwanted contribution to the autofocus camera image varies with defocus, it cannot be easily subtracted. To mitigate this, we replaced the single mode diode laser used in our previous autofocus system^5^ with a superluminescent diode (Superlum, S930‐B‐I‐10) that provides an autofocus beam that is spatially coherent but has a short (∼7 μm) temporal coherence length. Thus, only background signals that are back‐reflected from components within half the coherence length of the coverslip will produce an interference pattern at the autofocus camera and any back‐reflections from optical components in the autofocus system or from the other side of the coverslip will only contribute to a fixed background, which is subtracted from the autofocus camera images acquired as described above.

The software to control this optical autofocus system was written as a plug‐in for *μManager* 2.0, for which a screenshot of the GUI is presented in Supplementary Information. This plug‐in enables the user to define the desired in‐focus plane relative to the coverslip and to activate or disable continuous closed‐loop autofocus correction. Limits can be set to prevent the axial translation of the objective lens or sample beyond predetermined safe positions if the autofocus system were to fail. A calibration procedure can be run to create the look‐up table to associate the autofocus metric values (i.e., FWHM of the Fourier transform power spectrum of the intensity projections) with their respective defocus offset values. The background image is stored to be subtracted from the autofocus camera image. Threshold autofocus metric values can be set to define the regions in which the short‐range (single‐shot) closed‐loop autofocus can be used and the regions where the longer‐range (and multistep) autofocus correction should be used. When using the autofocus with an *openFrame*‐based fluorescence microscope, the fluorescence image data acquisition is managed by the *μManager* multidimensional image acquisition plug‐in, while the autofocus module runs in parallel with a *μManager* autofocus device adapter that obtains live defocus values (via a socket) that are calculated by a Python programme (designated ‘*DefocusCalc*’) that communicates with the *μManager* autofocus device adapter. Thus, the operation of the autofocus module does not significantly increase the fluorescence image data acquisition time. When used in closed‐loop operation, the μ*Manager* autofocus device adapter determines the defocus approximately every 0.25 s and adjusts the *z*‐drive to bring the sample back into focus. If desired, the record of defocus measurements (*z*‐drift) during an experiment can be saved.

## 4 EVALUATION OF DUAL‐AXIS OPTICAL AUTOFOCUS

The initial defocus range that the autofocus can accommodate is limited by the signal to noise ratio (S/N) of the defocus metric plots (red and blue curves in Figure 3). Where the higher precision (red) curve provides sufficient S/N, that is, ±37.5 μm, the autofocus system can operate in single‐shot mode, providing rapid focus lock if required. Outside this range of focus lock, the longer‐range autofocus can operate over ±68 μm but can no longer directly determine the sign of defocus. Instead, the system can acquire two autofocus camera images with the relative sample‐objective lens position being changed by a few microns between them and then determine the sign and magnitude of defocus. Although less precise, this long‐range autofocus can move the system back into the range of the more precise (single‐shot) autofocus operation.

To determine these operating ranges of the short‐ and long‐range autofocus functions, the performance of this cylindrical lens‐based autofocus system was quantified by an automated image acquisition of 100 nm diameter TetraSpeck fluorescent beads (TetraSpeck, T727) arrayed in a 96‐well plate (Brooks Life Science Systems, MGB096‐1‐2‐LG‐L) in which each well was precoated with poly‐L‐lysine and filled with Type 1 water. The wells were traversed in a snake‐like pattern, moving row by row and acquiring a fluorescence image *z*‐stack for one FOV in each well over an axial range of ±4 μm relative to the focus position with axial sampling of 100 nm. At each new well, the change in defocus was sufficiently small that the single‐shot, high‐precision autofocus could be first applied to return the microscope to focus and then the fluorescence image *z*‐stacks were acquired. If the autofocus system performed perfectly, then the in‐focus image within the fluorescence image *z*‐stack (i.e., the central plane in the z‐stack) should be at the same axial coordinate as the initial in‐focus image for that FOV. To quantify this performance, the fluorescence z‐stack image data were analysed using *PSFj*,^27^ previously published software that considers all the individual 100 nm beads appearing in the FOV and determines the slice in the *z*‐stack for which each specific bead is best in focus. For each FOV, a histogram of the defocus values (i.e., offset of the bead in‐focus plane relative to the predicted in‐focus plane) can be generated and quantified by the mean and standard deviation of this distribution.

**FIGURE 4:**
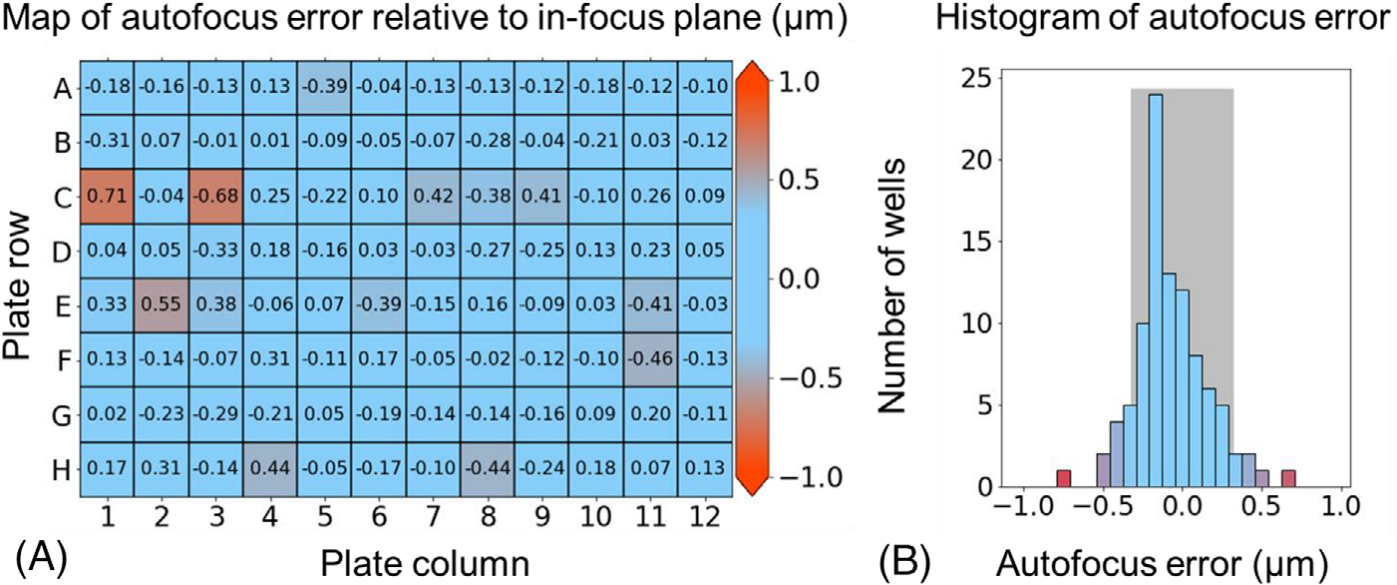
(A) A 96‐well plate arrayed with 100 nm TetraSpeck beads that was imaged using the autofocus with a *z*‐stack acquired in each well. The software package *PSFJ* was used to determine the mean ‘error’ (defocus) of the imaged beads (i.e., mean axial distance of all the in‐focus bead images in a FOV from the focal plane predicted by the autofocus) for each well is shown overlaid on a heatmap of this mean defocus in microns. (B) A histogram of mean defocus across the 96‐well plate (the depth of field for this fluorescence microscope with 100×, 1.4 NA objective lens was ∼0.65 μm for an emission wavelength of 680 nm – shown as grey shading).

Figure 4A presents the performance of the autofocus for each well as a colour map of the mean defocus (i.e., the mean offset between the autofocus‐predicted focal plane and the bead in‐focus plane within the *z*‐stack as determined by PSFj for each bead image) and also displays the numerical value of the mean defocus of the beads for each FOV. The colour scale indicates the variation of this mean defocus from 0 μm (blue) up to ±1 μm (red). Figure 4B presents a histogram of these mean defocus values/FOV, for which the standard deviation for the 96‐well plate is 230 nm and mean of the absolute prediction errors is 179 nm. The range of defocus values may be partly due to the bottom of the multiwell plate not being flat and parallel to the focal plane. The offset of the peak of the histogram in Figure 4B may be partly due to the centre of the beads being at least 50 nm from the coverslip to which they are attached, noting that the autofocus was manually set to maintain the top of the coverslip in focus.

To further quantify the performance of this cylindrical lens‐based autofocus approach, the accuracy of defocus correction from predetermined defocus values was measured. This was done by studying the ability of the autofocus system to return the microscope to focus starting from different amounts of applied defocus. From each preset defocus position, the autofocus was run 10 times and the objective lens was moved to the predicted in‐focus position for each of the 10 repeats. To test the shorter‐range (smaller confocal parameter) autofocus mode, initial defocus values ranging between ±20 μm of defocus in axial steps of 2 μm were used. To test the longer‐range (larger confocal parameter) mode, initial defocus values ranging between ±60 μm in 10 μm steps were used. These ‘focus‐corrected’ objective lens axial positions were compared to the true in‐focus objective lens position as determined by acquiring an autofocus camera *z*‐stack and interpolating between frames to find the plane where the size of the focused laser beam was minimised in the fast (i.e., short confocal parameter) direction.

**FIGURE 5:**
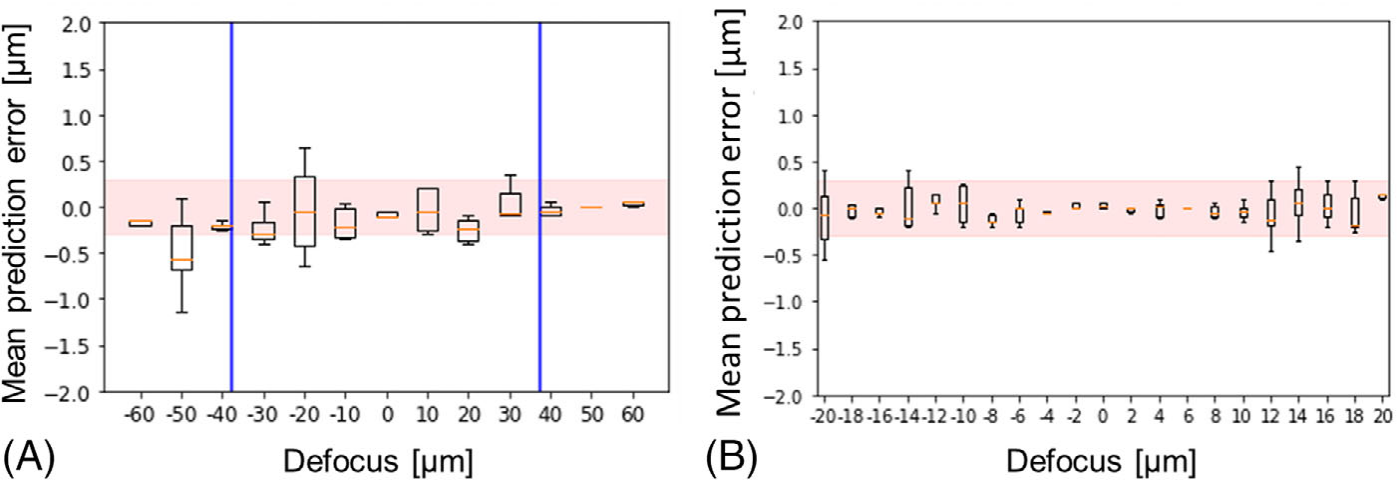
Errors in predicted defocus are plotted (boxplots with mean error, interquartile range and outliers). System was refocused 10 times from range of known defocus offsets. (A, B) Results for long‐ and short‐range autofocus respectively. Red highlighted region shows depth of field of microscope objective lens. Blue bars indicate the boundaries of the region where autofocus can operate in single‐shot mode.

The differences (errors) between the known defocus offset and the autofocus‐predicted defocus correction were calculated for each repeat measurement from each initial defocus offset value. Figure 5A and B show the mean and standard deviation of the defocus errors for the shorter‐range and longer‐range autofocus predictions respectively. As the initial defocus value was decreased, the error in defocus prediction tends to decrease as expected because the gradients of the autofocus metric curves (Figure 2) increase towards the peak. The autofocus can operate in single‐shot functionality over a range of ±37.5 μm (the region where the red plot in Figure 2 is above the noise level) and can operate as a two‐step process up to ±68 μm. When imaging the 96‐well plate we found the neighbouring well axial position variation to be less than 20 μm, so the accuracy within this region is shown in Figure 5B in finer defocus offset intervals of 2 μm. Within ±20 μm of focus, the autofocus could correct focus to within the depth of field and within ±5 μm of defocus, the accuracy of the defocus was better than 100 nm.

We also monitored the stability of this autofocus system over time by imaging a USAF 1951 test chart and using the decrease in the gradient of the edge of a test chart bar as a metric of defocus. As shown in Figure S4, the *openAF* autofocus system maintained the *openFrame* microscope in focus over 5 h within a displacement standard deviation of 35 nm, well within the depth of field of the 100×, 1.4 NA objective lens.

## 5 *easySTORM* ON AN openFrame MICROSCOPE

Using the components listed in Supplementary Information, we have implemented SMLM on an *openFrame*‐based microscope using *easySTORM* ^7^ – an implementation of SMLM that utilises multimode diode lasers and multimode optical fibres – with high average total power (>500 mW) uniform illumination exciting samples over FOV >120 μm diameter. Noting that the camera is a critical component for SMLM and often presents a significant cost, we present here the use of the CellCam Kikker (Cairn Research Ltd) CMOS camera for *openFrame*‐based *easySTORM*. We have previously compared the *easySTORM* performance of the CellCam Kikker CMOS camera used here with a sCMOS camera (Photometrics Prime 95B) on a commercial microscope frame and obtained similar performance (unpublished), as shown in Figure S3.

Since the CellCam Kikker provides a full‐frame imaging rate up to 17 fps for 14‐bit readout (with 4.63 μm pixel size), which is lower than is desirable for STORM at the excitation power levels (0.5–4 kW/cm^2^) available from our multimode diode lasers, we typically increase the frame rate by demagnifying the fluorescence image onto the camera by 0.35× and limiting the image data readout to the corresponding subregion of the camera CMOS sensor to reduce the image data size and increase the image frame rate to 33 fps – noting that the pixels after demagnification are still below the Nyquist limit (119 nm for emission at 670 nm) for our objective lens (Olympus UplanSApo 100× 1.40 oil). For this, we use a demagnifying C‐mount camera adapter (Motic, #1101001904111) that increases the effective size of the camera pixel to 115 nm in the sample plane. No significant vignetting of the demagnified images was observed.

We typically process the *easySTORM* image data using a parallelised implementation of *ThunderSTORM* running on an Imperial College HPC cluster^28^ or use PICASSO^29^ running on a desktop personal computer with a GPU (e.g., NVIDIA GeForce RTX 2080 SUPER).

To further test the performance of *easySTORM* microscopes, we have imaged nuclear pore complexes in the U‐2OS‐CRISPR‐NUP96‐SNAP cell line clone^30^ (distributed by CLS Cell lines Services GmbH) that is becoming a standard test sample for super‐resolved microscopy. This cell line expresses SNAP‐tag fused nucleoporin Nup96. To make this more widely useable, we have synthesised BG‐iFluor647, a new charged SNAP‐tag‐substrate to label nucleoporin Nup96, in order to reduce background from unbound or mis‐targeted fluorophores.^31^ In our hands, iFluor 647 is the most effective fluorophore we have used for dSTORM and has been reported to not require an enzymatic oxygen scavenger system in the imaging buffer such as the commonly used glucose oxidase‐catalase system,^32^ which is important for widening access to dSTORM, since these enzymes are not easily available in some countries. This extends the concept of Ref. (31) to yield a more convenient known biological test sample with low background, specific labelling and relatively simple sample preparation that we hope can improve access to SMLM workflows. We note that widening access to STORM by simplifying the sample preparation and using more easily available reagents is being addressed, including for multicolour STORM.^33^

**FIGURE 6:**
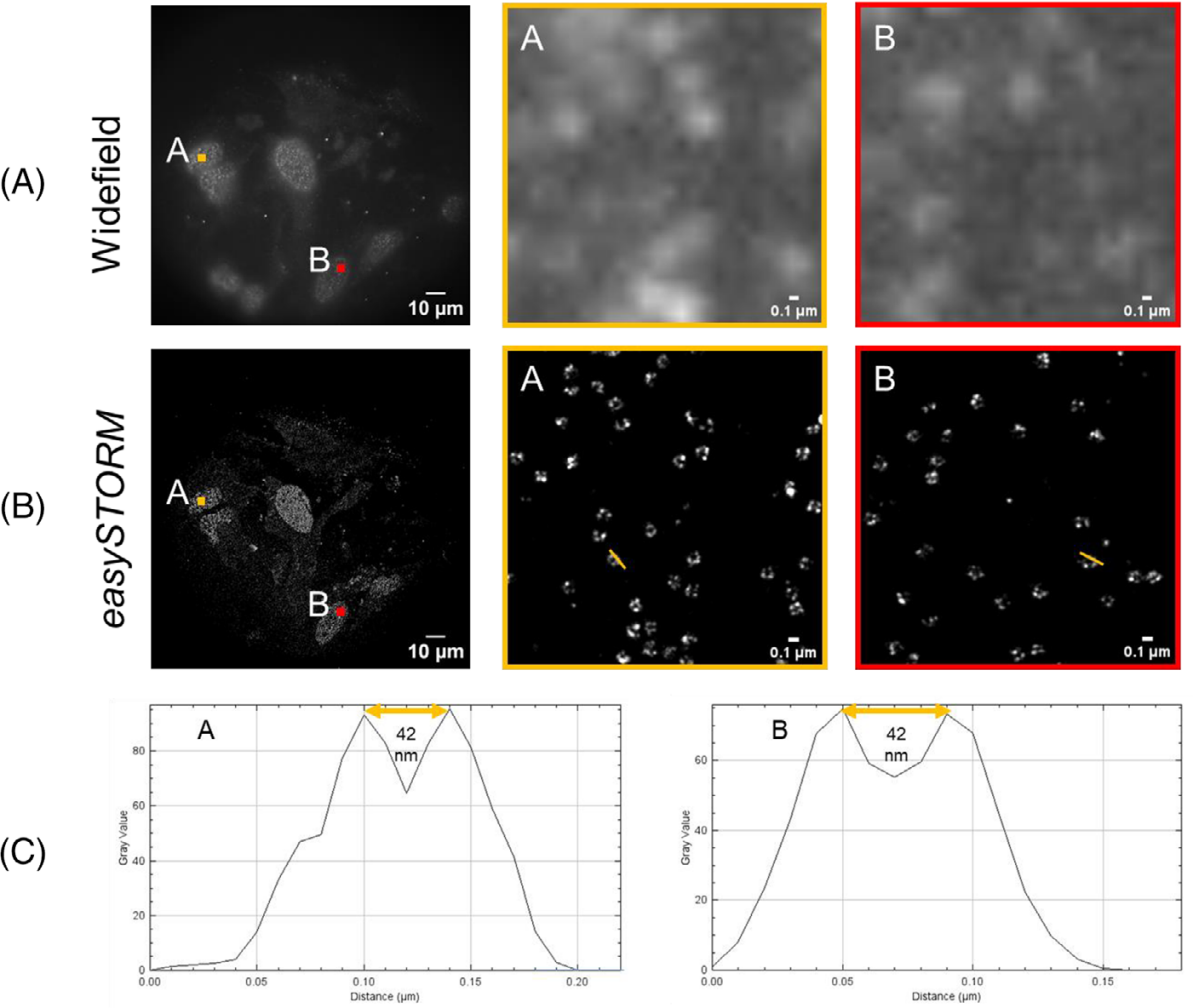
HILO wide‐field fluorescence (A) and reconstructed *easySTORM* (B) images of nucleopores in fixed U‐2OS‐CRISPR‐NUP96‐SNAP‐iFluor647 cells acquired on the *openFrame*‐based *easySTORM* microscope using cooled CMOS camera with continuous closed‐loop autofocus correction. Zoomed regions A and B, indicated in the full FOV images, are presented showing nucleoporin Nup96 proteins. (C) The line profiles through neighbouring Nup96 proteins as indicated in the zoomed easySTORM images. SMLM data was processed using PICASSO.^29^

This nuclear pore substructure is frequently imaged using SMLM implemented in a total internal reflection fluorescence (TIRF) microscope but, since we aim to widen access to SMLM through lower cost instruments, we avoided the expense of a TIRF objective lens and imaged this cell line using a standard (Olympus UplanSApo 100 × 1.40 oil) fluorescence objective lens in highly inclined and laminated optical sheet (HILO) mode.^34^ Figure 6 shows *easySTORM* images of these U‐2OS‐CRISPR‐NUP96‐SNAP‐iFluor647 cells, illustrating the ability to resolve substructure of the nuclear pores. Figure 6A and B shows the whole acquired field of view, with multiple nuclei present, while Figure 6C–F shows zoomed in regions indicated in Figure 6B. The clusters of nuclear pore Nup96 proteins separated by 42 nm in the ring structure when viewed along the axis of the pore^30^ are separated in the image, indicating a resolution significantly beyond the diffraction limit. See Supplementary Information for details of sample preparation and *easySTORM* imaging protocols. Note that, because the nuclear envelope is not flat or parallel to the coverslip, only a subset of the nucleopores in the HILO field of view will be in the focal plane of the microscope.

**FIGURE 7:**
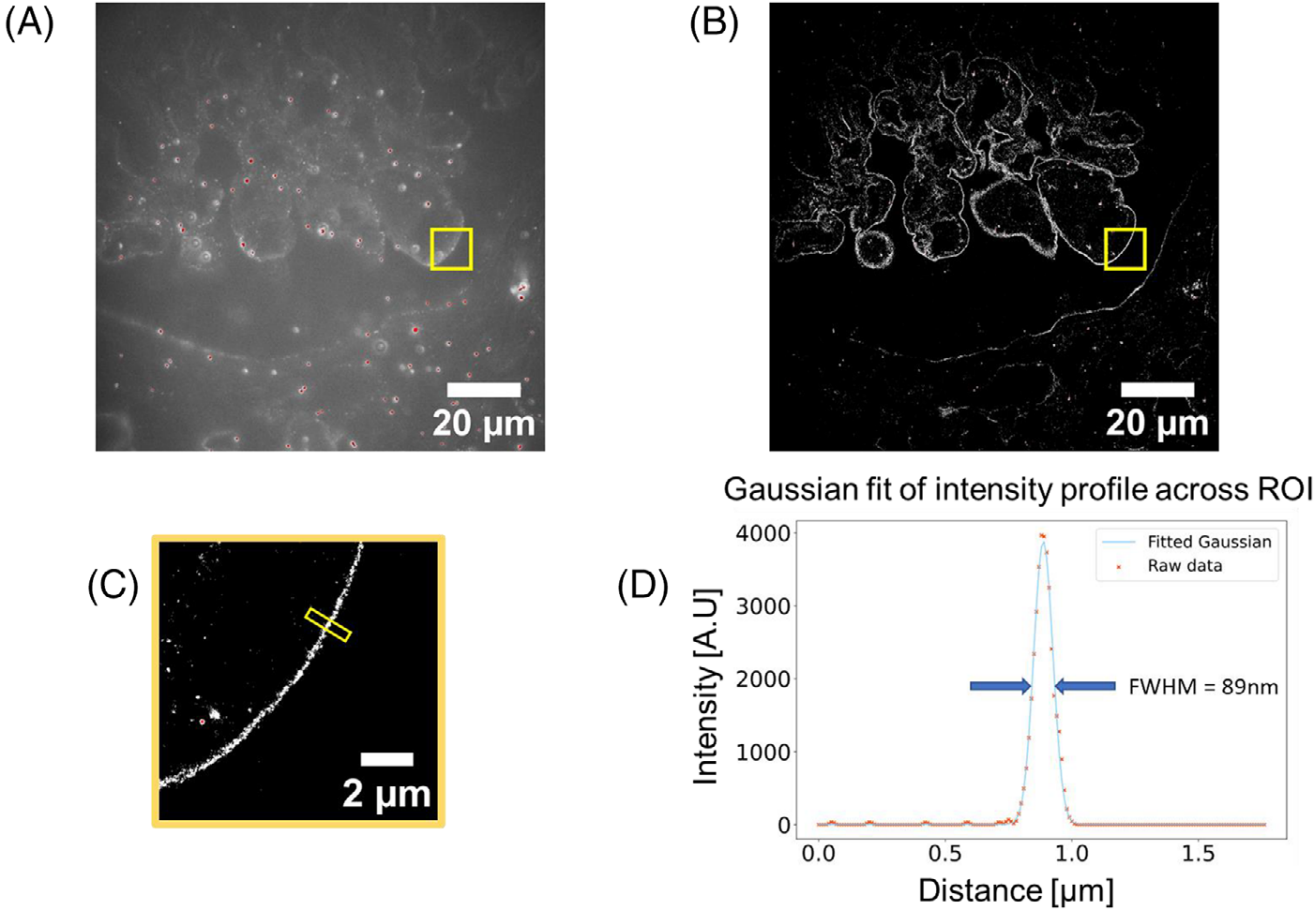
Epifluorescence (A) and STORM (B, C) images of an FFPE section of kidney biopsy tissue presenting lupus nephritis acquired on cost‐effective *openFrame*‐based microscope with cooled CMOS camera and continuous closed‐loop autofocus correction. (D) A section through the glomerular basement membrane indicating a thickness of 89 nm, which is in the same order of magnitude as expected when measured using electron microscopy. The bright features coloured red in (A) are 100 nm diameter TetraSpeck fluorescent microspheres that are added to the sample to provide fiduciary markers for lateral drift correction. SMLM data was processed using PICASSO.^29^

To illustrate a potential biomedical application of this *openFrame*‐based SMLM microscope, we repeat a previously reported experiment^35^ undertaken with *easySTORM* implemented on a commercial microscope frame (Carl Zeiss, Axiovert 200). Figure 7 shows the wide‐field epifluorescence and corresponding STORM images of a formalin fixed paraffin‐embedded (FFPE) histological section of kidney tissue from a patient biopsy presenting lupus nephritis (systemic lupus nephritis affecting the kidney), where the laminin in the glomerular basement membrane (GBM) is labelled with iFluor 647. This kidney section was prepared and imaged according to the ‘*histoSTORM*’ approach reported in our previous work.^35^

Figure 7 illustrates the ability of *openFrame*‐enabled *histoSTORM* to provide *easySTORM* images of kidney tissue structure and Figure 7D shows a line section through the STORM image of the laminin in the GBM, indicating the ability to measure the thicknesses of the glomerular basement membrane significantly below the diffraction limit. As discussed in Ref. (35), electron microscopy is used in UK clinical practice to diagnose kidney disease because conventional light microscopy cannot provide sufficient resolution, but SMLM could potentially enhance the diagnosis of kidney disease where electron microscopy is not available. A relatively low‐cost *openFrame*‐based microscope could provide *histoSTORM* as well as bright‐field and conventional immunofluorescence imaging of standard clinical histological sections.

## 6 CONCLUSIONS

We have introduced the modular *openFrame* microscope stand that can be used to develop cost‐effective, research‐grade microscopes, including for SMLM. The ability to utilise open‐source software and self‐built hardware components enables the assembly and maintenance of instruments at relatively low cost and the ability to rapidly configure new instruments can help accelerate the prototyping of new microscopes. Since an *openFrame*‐based microscope can easily be adapted or upgraded, or its components can be recycled, we hope to avoid obsolescence. A key component for advanced optical microscopes, including automated microscopes for high content analysis, is a hardware‐based autofocus. In general, we find the performance of *openFrame*‐based microscopes to be similar to that of research instruments in our laboratory that are configured with commercial microscope frames (e.g., Olympus IX81). Taking advantage of the flexibility in changing the optical/optomechanical configurations, we are now using *openFrame*‐based microscopes for many of our current research projects. Hence, we describe *openFrame*‐based microscopes as ‘research‐grade’. A simple *openFrame*‐based bright‐field or fluorescence microscope system can be configured using purchased components for <£10,000 (depending on configuration) and the approximate costs to assemble a motorised (*x*–*y*–*z*) *easySTORM* microscope with multiple excitation lasers and transillumination is £35,000 (excluding computer), as outlined in Supplementary Information.

We also present here a novel design for a robust optical autofocus system that can be added to an existing optical microscope, including an *openFrame*‐based instrument. This design addresses the trade‐off between autofocus precision and range through the use of cylindrical lenses to adjust the confocal parameter of the autofocus laser beam in orthogonal directions. We have implemented a calibration‐based look‐up table to determine the displacement of the coverslip from the focal plane of the objective lens and shown that this two‐step process works over a range of ±68 μm with an accuracy of ∼230 nm, which is well within the depth of field of the 1.4 NA objective lens. This approach also provides single‐shot autofocussing capability by determining the sign of defocus within the range of the shorter autofocus confocal parameter (±37.5 μm), thereby enabling closed‐loop ‘real‐time’ focus lock. In this mode, it can maintain focus at the coverslip to within 50 nm of the focal plane over many hours. Unlike our previous design, this new approach does not require machine learning but could still benefit from the approach outlined in Ref. (5), which may extend the operating range as the machine learning can work with a lower signal to noise ratio if needed. Although we developed this autofocus module to use on *openFrame*‐based microscopes, it can be implemented on other microscope frames provided that the infrared autofocus laser beam can be introduced into the system and reflected off the microscope coverslip. For example, we have implemented a version of this autofocus module with an Olympus IX81 fluorescence microscope using an appropriate multiline dichroic beamsplitter to reflect the autofocus laser beam to the coverslip in line with the excitation laser beam. For this, we controlled the microscope *z*‐stage with our *μManager* autofocus plug‐in and the manufacturer's device adapter for the microscope frame's inbuilt *z*‐drive. We note that our Python programme calculating defocus runs independently of *μManager* and so could be used with other microscope control software.

To illustrate the performance of the *openFrame* microscope stand, we have presented exemplar *easySTORM* images of the nucleopore structure in U‐2OS cells where Nup96‐labelled subunits known to be separated by 42 nm can be seen to be resolved. We note that we are not claiming that the implementation of *easySTORM* used here provides the best performance possible with this microscope stand and objective lens – better results could potentially be realised using more powerful excitation lasers and higher frame rate scientific cameras^36^, ^37^ – but our implementation using low‐cost components – specifically the ∼700 mW power multimode laser diode and the CellCam Kikker CMOS camera provides reasonable performance at a level of cost and complexity appropriate to widen access to SMLM.

Following our earlier work,^35^ we also demonstrated easySTORM of ultrastructure in a clinical kidney tissue section using the *openFrame*‐based microscope, where the thickness of the GBM can be measured to a precision beyond the diffraction limit, potentially providing useful clinical diagnostic information beyond conventional immunofluorescence microscopy. The additional costs and data acquisition times (∼40 min to acquire an easySTORM image data set of 80,000 frames) compared to standard immunofluorescence is significant, but less than the costs and timescales of diagnostic electron microscopy, which is not available in many countries. As previously discussed,^7^ an existing fluorescence microscope can be upgraded to *easySTORM*. Depending on the existing microscope configuration, this may be undertaken for a component cost of less than £10,000 excluding computer and objective lens and not including an autofocus module.

## Supporting information

Figure S1

Figure S2

Figure S3

Figure S4

Supporting information 1

Supporting information 2
