# Supplementary figures and images for: openFrame: A modular, sustainable, open microscopy platform with single‐shot, dual‐axis optical autofocus module providing high precision and long range of operation
Source: J Microsc. 2023 Sep 27;292(2):64–77. doi: 10.1111/jmi.13219 (PMC10953376; doi:10.1111/jmi.13219)

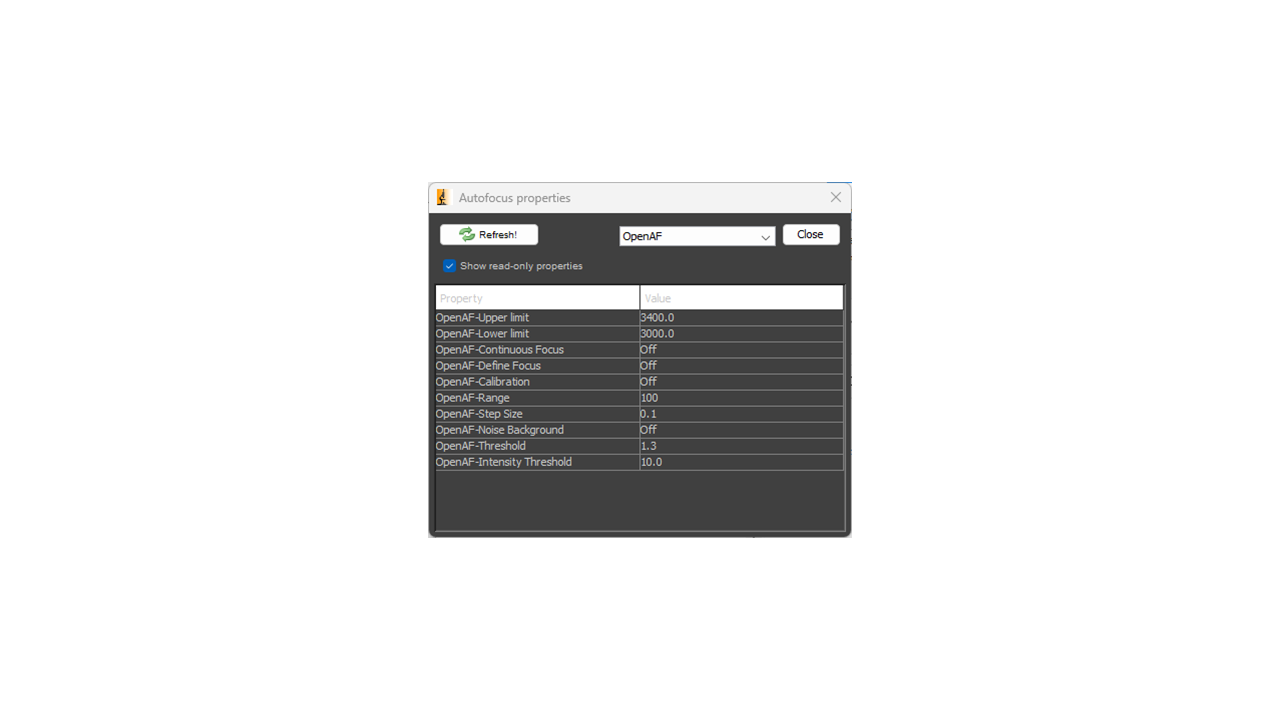

Supplement: Supplementary file 1 — Figure S1 [file JMI-292-64-s003.png]

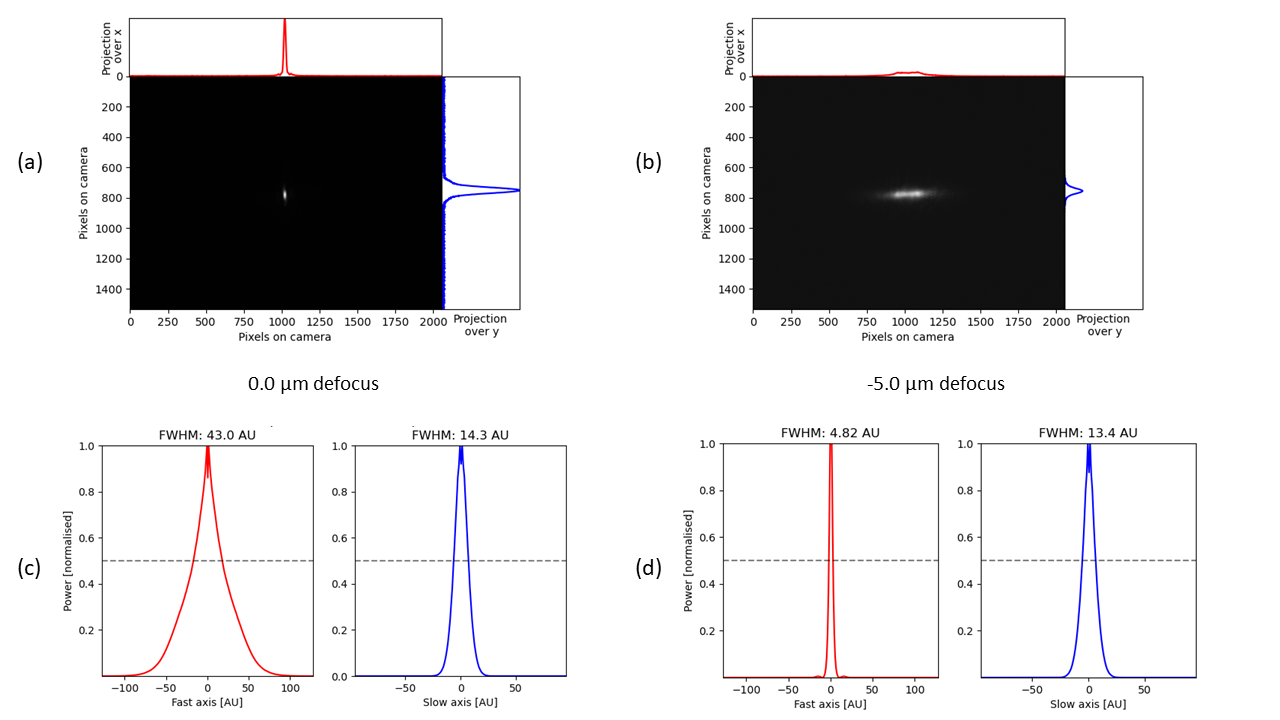

Supplement: Supplementary file 2 — Figure S2 [file JMI-292-64-s004.png]

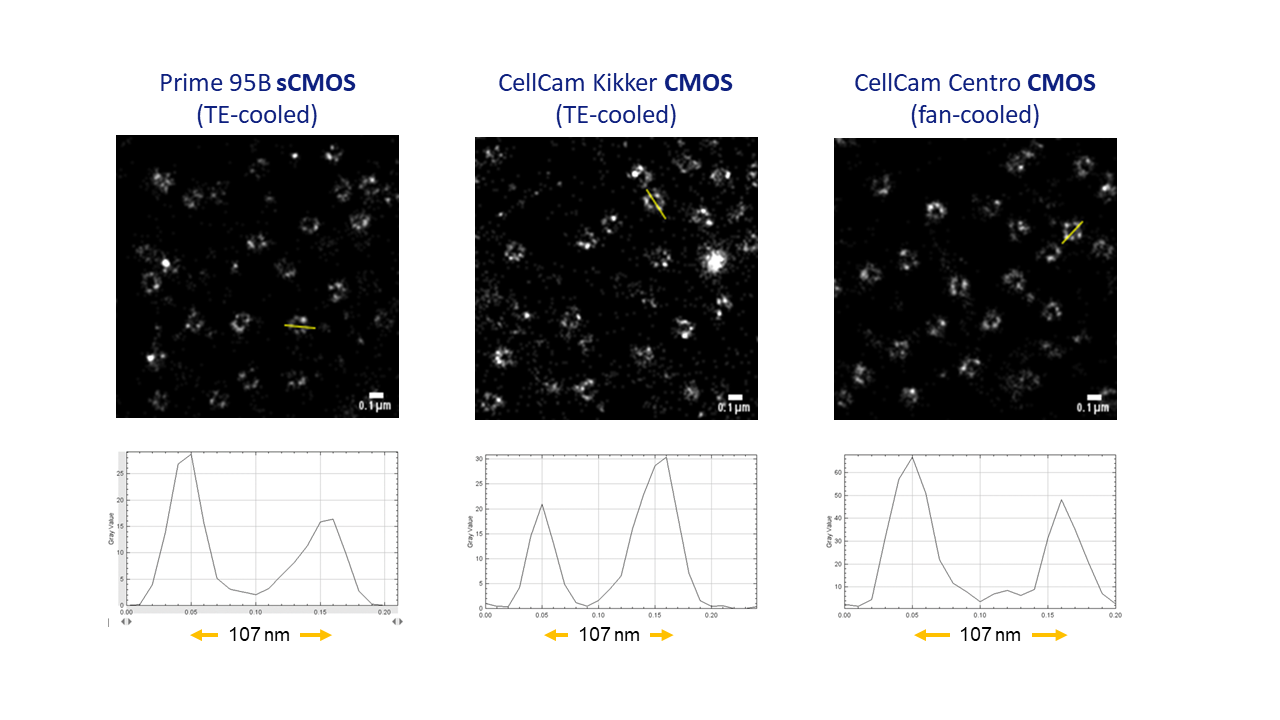

Supplement: Supplementary file 3 — Figure S3 [file JMI-292-64-s001.png]

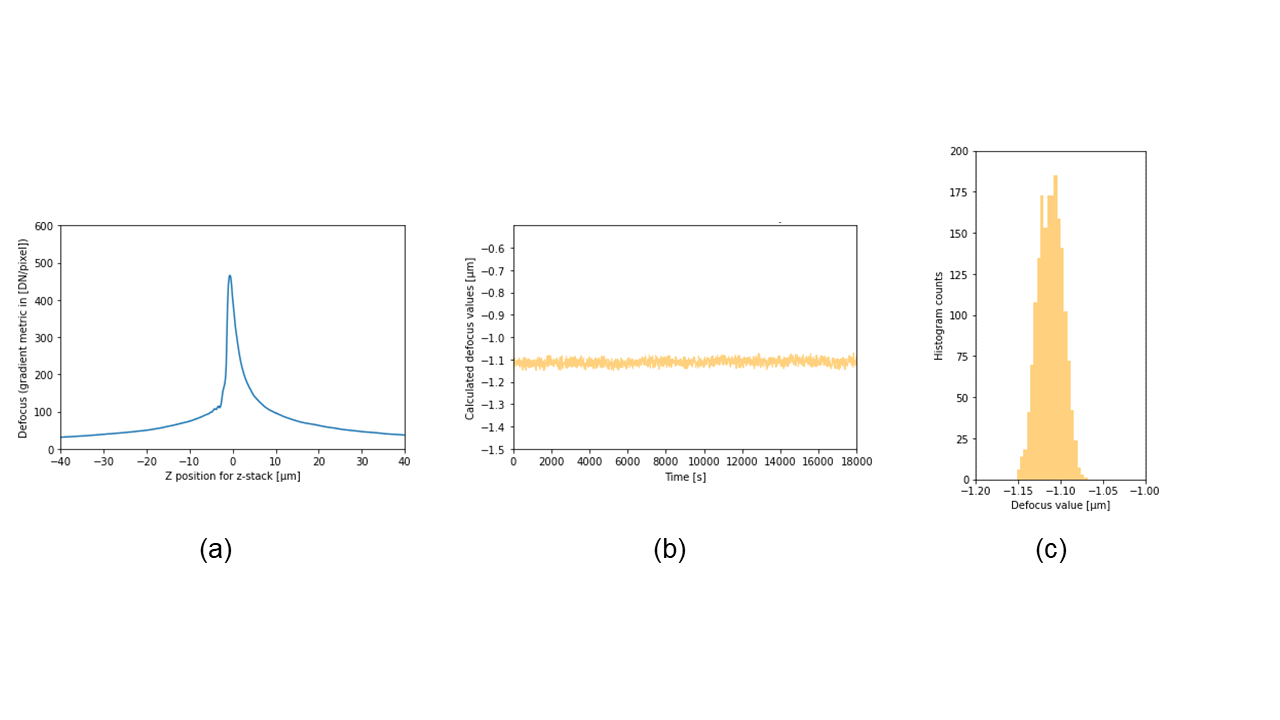

Supplement: Supplementary file 4 — Figure S4 [file JMI-292-64-s002.png]
