# Supplementary material for: openFrame: A modular, sustainable, open microscopy platform with single‐shot, dual‐axis optical autofocus module providing high precision and long range of operation
Source: J Microsc. 2023 Sep 27;292(2):64–77. doi: 10.1111/jmi.13219 (PMC10953376; doi:10.1111/jmi.13219)
Supplement: Supplementary file 5 — Supporting information 1 [file JMI-292-64-s006.docx]

*openFrame*: a modular, sustainable, open microscopy platform with single-shot, dual-axis optical autofocus module providing high precision and long range of operation.

J. Lightley, S. Kumar et al.,

**SUPPLEMENTARY INFORMATION**

**Components for *openFrame*-based *easySTORM* microscope.**

The *openFrame* concept originated at Imperial College London and has been co-developed with Cairn Research Ltd (and sister company, Cairn GmbH). The *openAF* optical autofocus module was developed at Imperial College London. Information to support the assembly and use of *openFrame*-based microscopes and *openAF* modules should be available at: <https://www.imperial.ac.uk/photonics/research/biophotonics/instruments--software/fluorescence-microscopy/openframe/>. The *openFrame* microscope reported in this paper - and similar instruments - can be constructed using widely available commercial components and/or self-fabricated core *openFrame* components for which we are sharing CAD files. In general, *openFrame*-based microscopes have been designed to be controllable with open-source software such as [*µManager*](https://micro-manager.org/). Cairn Research Ltd will also sell these core *openFrame* components to users who wish to assemble their own instruments but not to fabricate the components themselves. In addition, Cairn designs and sells further *openFrame*-compatible components and bespoke application-based *openFrame* microscope solutions combining open-source parts with proprietary Cairn and third-party components.

We are licensing the core *openFrame* components and the *openAF* optical autofocus module under under the permissive version of the CERN Open Hardware License Version 2 (<https://ohwr.org/cern_ohl_p_v2.pdf>), including the component designs hosted on this repository. We do not wish to restrict users in their ability to utilise *openFrame* components and, as well as supporting the assembly of open instrumentation, *openFrame* components can be used with closed-source and proprietary technologies.

Hardware components

The parts list to build an *openFrame*-based easySTORM microscope is available at <https://github.com/ImperialCollegeLondon/openFrame>. The CAD files for fabrication of core *openFrame* components licensed under the permissive version of the CERN Open Hardware License Version 2 can be found in the folder called “openFrame CAD”.

The parts list to build an *openAF* optical autofocus module similar to that used in this is available at <https://github.com/ImperialCollegeLondon/openAF>. The openAF optical autofocus module is primarily assembled from commercially available components. CAD files for any parts that are not commercially available can be found in the folder called “openAF CAD”. The design and CAD files for the openAF optical autofocus module are licensed under the permissive version of the CERN Open Hardware License Version 2.

Fabrication & assembly

Further information to support fabrication and/or assembly of an exemplar *openFrame*-based epifluorescence microscope can be found in the folder called “*openFrame* assembly instructions”. Please note that a wide range of configurations of *openFrame*-based microscopes are possible, and only one exemplar instrument is outlined here. Further information to support assembly and operation of an *openAF* optical autofocus module can be found in the folder called “openAF assembly instructions”.

Software components

We make extensive use of [*µManager*](https://micro-manager.org/) to control instrumentation but understand that this may not always be the first choice of all users or developers. µManager uses the Lesser GPL license (<https://www.gnu.org/licenses/lgpl-3.0.en.html>) for its core and the BSD 2-clause license for the GUI and device adapters, and any µManager device adapters we have written (for the *openFrame* hardware components licensed under the permissive version of the CERN Open Hardware License Version 2 in this repository) are licensed under the BSD 2-clause license.

Any other software for the open-source components *openFrame* hardware components licensed under the permissive version of the CERN Open Hardware License Version 2 in this repository is also licensed under the BSD 2-clause license (<https://opensource.org/license/bsd-2-clause/>).

The *openFrame* microscope is controlled using µManager and we use the standard Multidimensional Data acquisition (MDA) plugin to control the *openFrame*-based microscope image acquisition, including when undertaking *easySTORM*. We use device adapters included with the standard µManager distribution for some commercially available components, including the piezo actuator/controller (PI, Q-545.140 and PI, E-873.1AT controller) for z-scanning the objective lens, the imaging camera (Cairn, CellCam Kikker and CellCam Centro) and the autofocus camera (FLIR, CM3-U3-31S4M-CS) and the XY stage (Zaber, ASR100B120B). We have written a µManager plugin to control the *openAF* autofocus module (screenshot shown below) that is licensed under the BSD 2-clause license (<https://opensource.org/license/bsd-2-clause/>).

The *µManager* *openAF* autofocus plugin interface shown in Supplementary Figure 1. The Python code for running the *openAF “**DefocusCalc”* module is licensed under the BSD 2-clause license (<https://opensource.org/license/bsd-2-clause/>).

The *easySTORM* SMLM data was processed using ThunderSTORM implemented on a high-performance computing cluster [28] or PICASSO [29]. Other image data processing was undertaken using ImageJ [3] and plugins for it such as PSFJ [27].

*Supplementary Figure 1. GUI for µManager openAF autofocus plugin.*

Acknowledgements

The *openFrame* is a compact, modular, open-source microscope conceived by Paul French and developed with colleagues from the [Photonics Group at Imperial College London](https://www.imperial.ac.uk/photonics/research/biophotonics/instruments--software/fluorescence-microscopy/openframe/), and then designed, supported, and extended in collaboration with Cairn Research. The first prototype *openFrame* microscope was co-designed and fabricated by Simon Johnson and Martin Kehoe in the Optics instrumentation facility of the Physics Department at Imperial College London. Subsequently the design and implementation were refined in a collaboration with Cairn Research Ltd by Frederik Görlitz and Sunil Kumar at Imperial supervised by Paul French, working with Callum Hollick at Cairn supervised by Jeremy Graham. Callum Hollick fabricated subsequent prototypes and designed the current version of core *openFrame* components, which are represented in these CAD files.

The *openAF* approach to optical autofocus using either a slit or orthogonal (offset) cylindrical lenses to simultaneously provide short- and long-range operation with different confocal parameters was conceived by Paul French and developed with colleagues from the Photonics Group at Imperial College London. Frederik Görlitz and Jonathan Lightley, supervised by Paul French and with input from Sunil Kumar, Christopher Dunsby, Seth Flaxman and Mark Neil, implemented the first (CNN-based) version of our optical autofocus [5] using a rectangular slit in the collimated autofocus laser beam to set the different orthogonal confocal parameters and wrote the associated software, which included the use of machine learning to analyse the autofocus camera images. Subsequently, Jonathan Lightley, working with Sunil Kumar and Paul French and with input from Christopher Dunsby and Mark Neil, implemented the current (*openAF)* version of the autofocus system that does not utilise machine learning and uses orthogonal offset cylindrical lenses to collimate the autofocus laser beam from a superluminescent diode.

**Supplementary Methods**


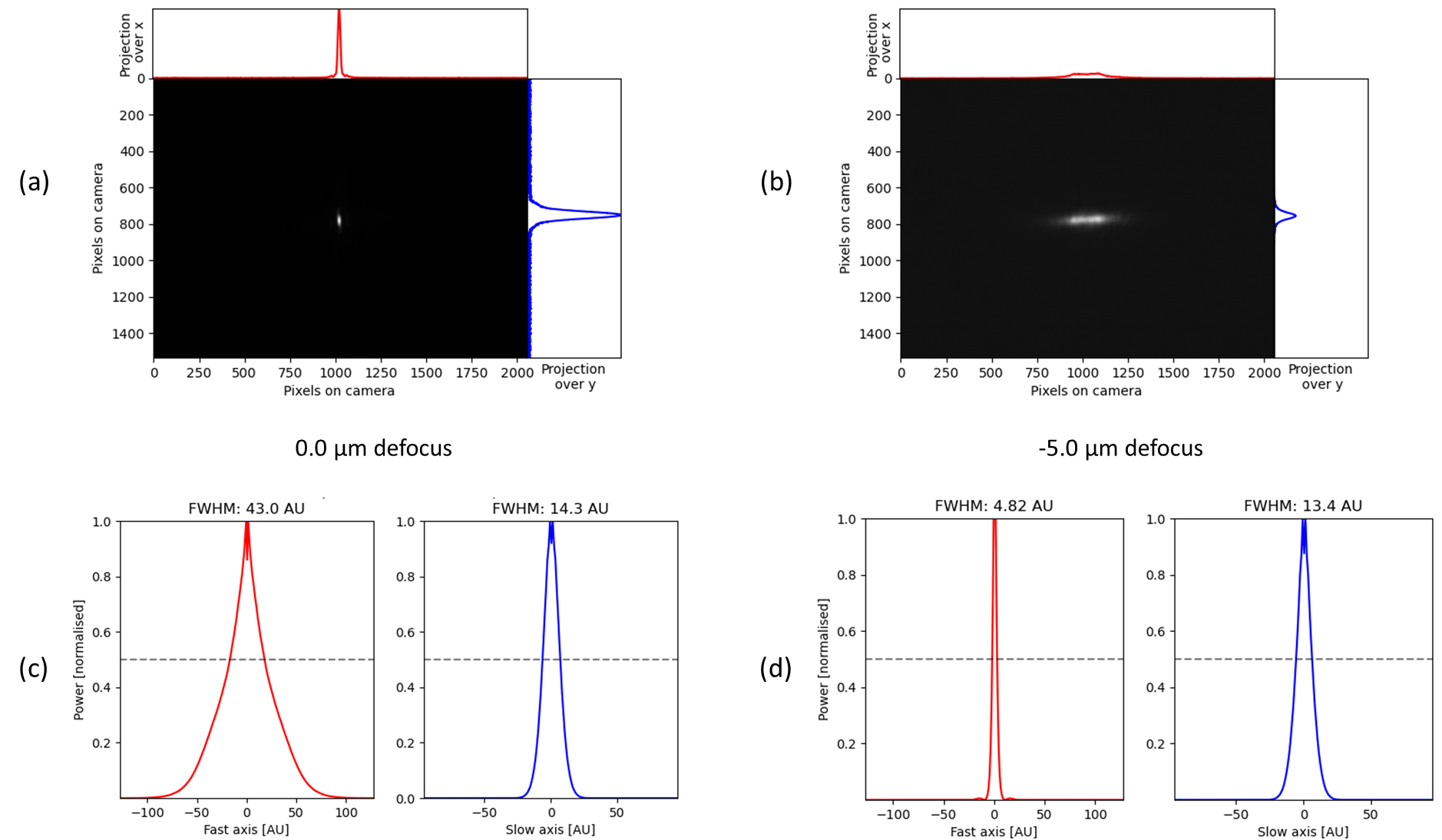


*Supplementary Figure 2. (a, b) shows the background-subtracted autofocus camera images and the mean intensity projections recorded with the sample coverslip at axial positions 0.0 µm and -5.0µm from the focal plane respectively with (c, d) showing the corresponding Fourier power spectrum line profiles.*

Calculation of defocus metric

Supplementary Figure 2 illustrates how the defocus metric is calculated for two specific values of defocus (indicated on Figure 3). Briefly, when the defocus is checked by the Python programme “*DefocusCalc*”, a background-subtracted autofocus camera image is binned along each axis (parallel to the shorter and longer focal length cylindrical lenses to provide projection line profiles as inputs for calculation of the long- and short-range confocal parameter defocus metrics. An autofocus background image is recorded before each imaging session, which is the mean of two images acquired with the sample (coverslip) being translated 100 µm above and below the focal plane. This background image is subtracted from all subsequent autofocus camera images. The intensity projection line profiles are Fourier transformed to provide the Fourier power spectra and the respective defocus metrics are given by the FWHM of these power spectra. Calibration curves are established by axially scanning a (coverslip) sample through focus and the resulting defocus metrics are plotted as a function of z, as shown in Figure 3. The mean autofocus camera pixel intensity is also calculated for the background subtracted image. Going forward, real-time defocus metrics for the long-and short-range confocal parameters can then be calculated from an autofocus camera image. First the total intensity is checked (against the green mean intensity curve in Figure 3) to determine if is within the usable range of the short confocal parameter defocus metric (and therefore the closed-loop single shot operation, i.e., ±37.5 µm). If it is (as is usually the case), the objective lens is moved directly to restore focus. If not (e.g., because microscope has moved to a different field of view in a multiwell plate), then the system operates a 2-step process, recording two autofocus camera images separated by an axial displacement of 20 µm to determine the magnitude and sign of defocus (calculating the long confocal parameter defocus metric for each image) and then the objective lens is moved to be close to focus. Once near focus, the system operates in closed loop with <50 nm displacement error (See Figure 5 and Supplementary Figure 4).

HILO easySTORM imaging protocol

For HILO illumination of the sample, the beam-steering mirror in the excitation path (M1 in Figure 1) was used to adjust the position of the focussed excitation laser beam close to the periphery of the back focal plane of the objective lens, such that the fiducial markers were visible across the field of view. Suitable FOVs for imaging were identified using an illumination intensity at the sample plane set to 0.02 kW.cm^-2^ with a camera integration time of 30 ms. The sample was manually surveyed to locate suitable regions (e.g., cells exhibiting a nuclear membrane sufficiently close to the sample interface) for HILO illumination, ensuring that at least 2 fiducial markers are visible in the imaging FOV. Once a suitable FOV was located, a widefield fluorescence image was acquired (using 635 nm laser radiation with 0.04 kW.cm^-2^ intensity at the sample) with an integration time of 2000 ms. For *easySTORM*, the fluorophores were driven into the dark state using 635 nm laser radiation with 0.5 kW.cm^-2^ intensity at the sample) until the distribution of blinking fluorophores was determined to be sufficiently sparse for SMLM. Then *easySTORM* data was acquired (typically for 40,000 -80,000 frames), using the *µ-Manager* multidimensional acquisition plug-in. Over the course of the acquisition, the frequency of blinking in the sample was monitored, and a 405 nm multimode diode laser was used to enhance switching of molecules out of the dark state (with the intensity at the sample being increased in steps typically every 5-10k frames from zero by 0.005 kW.cm^-2^ per step, up to a final power of 0.028 kW.cm^-2^.

Using the CellCam Kikker CMOS camera with the 0.35x demagnifying C-mount camera adapter (Motic, #1101001904111), the pixel size (115 nm in the image plane) is sufficient for Nyquist sampling of the expected PSF width for a peak emission wavelength of 670 nm using a 1.4 NA objective without aberrations.

Preparation and easySTORM of nucleopore test samples

We used the osteosarcoma clonal stable cell line generated by CRISPR-Cas9D10A nickase-assisted genome editing (U-2 OS-Nup96-SNAP clone no.33 cells) distributed by Cell Line Services, GmbH Catalogue No. 300444). This cell line [25] is tagged at Nucleoporin 96 (NCBI accession Number: NM_016320, (<https://www.ncbi.nlm.nih.gov/nuccore/NM_016320.5/>) with a SNAP-tag. The cells were cultured following supplier recommendations in McCoy’s 5A Medium (Merck, M4892) supplemented with 10% FBS, 1% glutamine, 1% penicillin-streptomycin, non-essential amino acids (Gibco 11140-050) and 36 mM Sodium bicarbonate (Gibco, 25080-094).

The following protocol was used to prepare the samples for *easySTORM*:

1. U-2 OS-Nup96-SNAP cells were seeded in 8 well Nunc™ Lab-Tek™ Chambered Coverglass (Thermo Scientific, 155411). The volume of liquid applied to each chamber slide well was 0.4 milliliters unless otherwise specified.
2. Cells were washed twice in Phosphate Buffer Saline (PBS) then fixed in 2.4% paraformaldehyde (PFA) in transport buffer (TRB) consisting of 20 mM HEPES, 1 mM EGTA, 10 mM KOAc, 5 mM Mg(OAc)_2_, 10 mM Sucrose)
3. Cells were then washed twice for 5 minutes in TRB without PFA followed by the application of 100 mM NH4Cl diluted in PBS for 5 minutes to quench autofluorescence of the PFA fixative, followed by two washes in PBS.
4. Cells were treated with four drops per chamber of Image-iT FX Signal Enhancer (ThermoFisher Scientific) for 30 minutes to block non-specific association of dyes due to negative charge.
5. Blocking buffer (Bovine Serum Albumin 3% in PBS with 50 mM Mercaptamine) was applied for 10 minutes to block unspecific binding to glass.
6. Cells were treated with *O*^6^-benzylguanine SNAPtag-substrate conjugated to iFluor 647® [26] at a concentration of 0.1 μM in blocking buffer overnight at 4°C to label the Nucleoporin 96 proteins.
7. Unbound SNAPtag-substrate removal with three five-minute washes with PBS-50 mM Mercaptamine
8. Each well of the chamber slide was treated with a 1:100 dilution of poly-L-lysine in PBS-50mM Mercaptamine for 5 minutes, then washed three times with PBS-50mM Mercaptamine.
9. Each well of the chamber slide was treated with a dilution 1:1000 of 0.1 μm TetraSpeck microspheres (ThermoFisher Scientific, T7279) for 10 minutes at room temperature.
10. Removal of unbound beads with three washes with PBS-50 mM Mercaptamine.
11. Sample pre-equilibration in STORM buffer (50mM Mercaptamine and 10mM Lactate in PBS) for 1 hour at room temperature.
12. Cells were imaged in fresh STORM buffer with 10 μL EC-oxyrase (Sigma Aldrich, SAE0010) in a parafilm sealed chamber slide.

For the data presented in Figure 6, this sample was activated with laser radiation at 635 nm at an intensity of 0.5kW.cm^-2^ and imaged with an intensity of 0.35 kW.cm^-2^ at the sample. 80,000 frames were acquired using the CellCam Kikker (Cairn Research Ltd,) CMOS camera with 30 ms exposures at 33 frames/s using the 0.35x demagnifying C-mount camera adapter (Motic, #1101001904111) to reduce the field of view such that the effective size of the camera pixel was 115 nm. We note that we used the Olympus UplanSApo 100x 1.40 oil) objective lens, which is designed for an Olympus tube lens of 180 mm focal length, with the (Nikon MXA20696) tube lens in the openFrame microscope that has a focal length of 200 mm. Thus, the effective magnification is 111x (or 38.9x using the 0.35x Motic demagnifier). The actual camera pixel size in image space when using the Motic demagnifier was measured to be 115 nm using a USAF 1951 test chart.

histoSTORM of FFPE kidney tissue section

The histological slide staining followed the *histoSTORM* protocol we previously reported [24]:

1. Paraffin removal by heating slides at 60^o^C for 10 minutes
2. Two xylene washes for 5 minutes each, followed by two-minute washes using decreasing concentration of ethanol diluted in water (100%, 75%, 50% 25% and 0%).
3. Antigen retrieval at 37^o^C for 27 minutes with Protease Type 24 (P8038 – Sigma-Aldrich) (0.125 mg/ml) in Phosphate Buffer Saline (PBS)
4. Autofluorescence quenching with Sodium Borohydride 1mg/ml in PBS for 10 minutes followed by three washes with PBS.
5. Blocking for 10 minutes in Blocking solution (3% BSA in PBS).
6. Application of primary antibodies at a dilution 1:10,000 Laminin MAB1920 (Millipore, Watford, Hertfordshire, UK) in Blocking solution and incubation for 20 minutes at room temperature.
7. Unbound primary antibody removal with three washes with PBS.
8. Application of secondary antibody conjugated with iFluor 647. Stock 0.25 mg/ml goat anti-mouse IgG H+L (16482; AAT Bioquest, Stratech, Ely, Cambridgeshire, UK) diluted at 1:2,000 in Blocking solution and incubation for 20 minutes at room temperature.
9. Unbound secondary removal with three washes with PBS.
10. Coating of slide with a 1:100 dilution of poly-L-lysine in PBS for 5 minutes, followed by three washes with PBS. Application of a dilution 1:1000 of TetraSpeck microspheres 0.1 μm (ThermoFisher Scientific, T7279) for 10 minutes at room temperature.
11. Removal of unbound beads with three washes with PBS.
12. Sample pre-equilibration in STORM buffer (50 mM Mercaptamine and 10 mM Lactate in PBS) for 1 hour at room temperature.
13. Mounting on slide of cover glass 22 x 22mm no1.5 high precision (Ref: 0107052 Marienfied, Würzburg, Germany) in fresh STORM buffer 0.4 milliliters with 10 μL EC-oxyrase (Sigma Aldrich, SAE0010).

For the data presented in Figure 7, this sample was activated and imaged with laser radiation at 635 nm with a sample intensity of 0.5 kW.cm^-2^. 80,000 frames were acquired using the CellCam Kikker (Cairn Research Ltd,) CMOS camera with 30 ms exposures at 33 frames/s using the 0.35x demagnifier (Motic) to reduce the field of view such that the effective size of the camera pixel was 115 nm.

*Supplementary Figure 3. Reconstruction of NUP96 proteins imaged using EasySTORM implemented on a (Carl Zeiss, Axiovert 200) using a Prime95B sCMOS, a CellCam Kikker 100MT and a CellCam Centro 200 MR camera* *All images acquired on the same day and reconstructed from 20,000 frames with 80 ms exposure.*

Comparison of cameras for *easySTORM*

Supplementary figure 2 shows the relative performance of the CellCam Centro (fan-cooled) CMOS camera and the CellCam Kikker (TE-cooled) CMOS camera relative to an established sCMOS camera (Photometrics Prime 95B) when used for easySTORM of U-2 OS-Nup96-SNAP clone no.33 cells expressing the Nup96 proteins labelled with Alexa 647 [31]. This data was acquired using laser radiation at 635 nm with an intensity of 1.3 kW/cm2 at the sample mounted in a commercial (Carl Zeiss, Axiovert 200) microscope using a TIRF (Zeiss, α Plan-apochromat 100x/1.46 oil DIC Vis) objective lens.

Stability of openFrame-based microscope with openAF autofocus system over time.

To check the stability of the openAF autofocus over time, we needed an independent real-time readout of defocus. We therefore imaged a USAF 1951 test chart mounted on the *openFrame* microscope using transmitted light and acquired a through-focus z-stack of images on the main microscope camera. We selected a region of interest (ROI) with a test chart bar and measured the gradient (between 75% and 15% of peak intensity in ROI) of the edge of the bar in each frame. Since this gradient decreases with increasing defocus (as the image of the test chart becomes less sharp), it provides a metric to quantify defocus. Supplementary figure 4(a) shows a plot of this gradient defocus metric as a function of measured axial displacement (z) through focus. This metric is monotonic either side of the central peak and can then be used as a look-up table to determine the defocus distance from any subsequent image of the test chart bar.

We acquired transmitted light images of the USAF 1951 test chart over 5 hours at 10 s intervals and Supplementary figure 4(b) plots the calculated defocus for each camera frame over the 5 hour acquisition time. Supplementary figure 4(c) presents a histogram of these defocus values which has a standard deviation of 35 nm, well within the depth of field of the of the 100x, 1.4 NA objective lens.


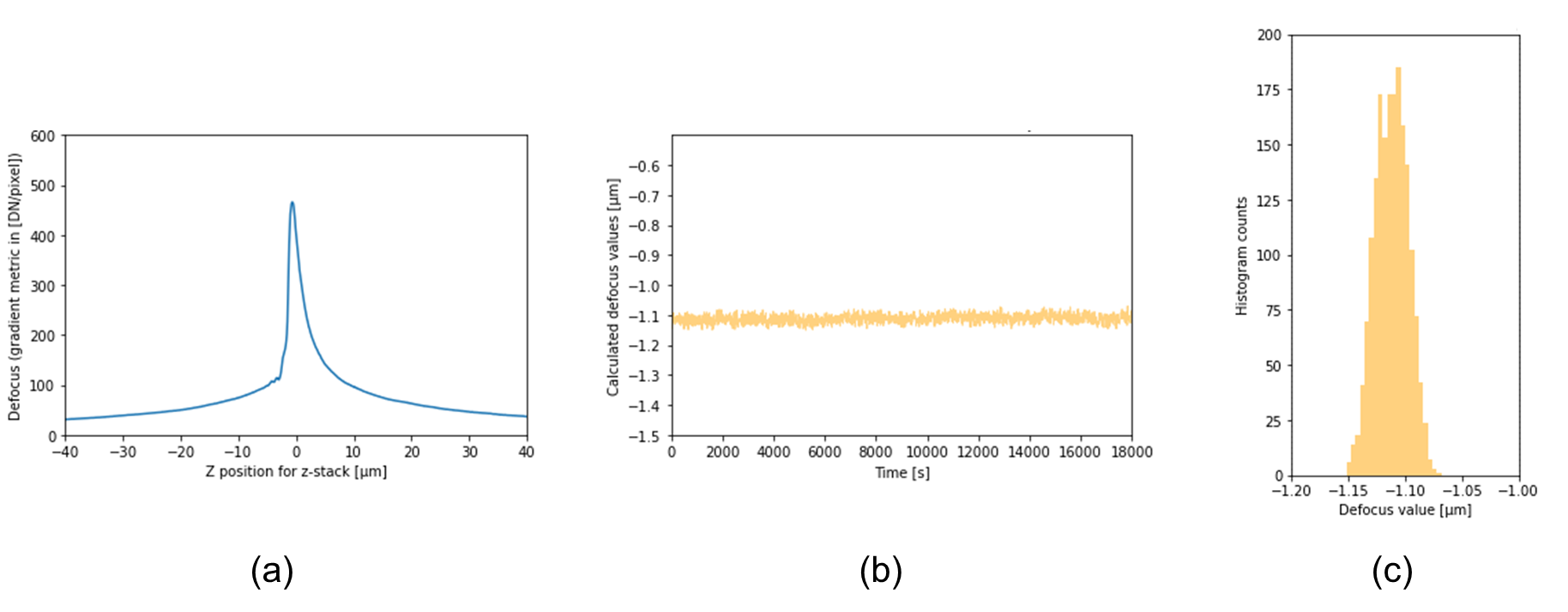


*Supplementary figure 4. (a) shows plot of focus metric (gradient of edge of test chart bar) as a function of z; (b) shows the variation over time (5 hours) of the sample defocus calculated using the gradient defocus metric; (c) histogram of defocus values from (b) with standard deviation of 35 nm.*

Approximate costs to assemble *openFrame* microscopes.

The cost of any *openFrame*-based microscope will depend on the functionality, Supplementary spreadsheet 1 presents the approximate costs of the components that can be used to assemble epifluorescence and *easySTORM* microscopes. Note that prices are subject to variation and many components/subsystems could be replaced by self-built alternatives, the following table summarises the figures in the spreadsheet to outline the components required to build up a motorised *easySTORM* microscope with motorised x-y stage and a piezo-electric z-drive.

|  |  | Price (£) |
| --- | --- | --- |
| *openFrame* components for epifluorescence microscope (excluding transillumination and objective lens) | Cairn Research Ltd | 6013 |
| Excitation components (excluding laser) | Thorlabs Ltd | 856 |
| Camera mounting components and tube lens | Cairn Research Ltd | 635 |
| CellCam Kikker 100MT (TE-cooled CMOS) camera with trigger box | Cairn Research Ltd | 3975 |
| CellCam Centro 200MR (fan-cooled CMOS) camera | Cairn Research Ltd | 1238 |
| Single fibre-coupled multimode diode excitation laser | Various | 784 |
| 4-colour fibre-coupled laser bank of multimode diode excitation lasers | Various | 4980 |
| Single line (GFP-FITC) filter set (including dichroic beamsplitter) | Chroma Technology | 744 |
| Multiline (405, 460, 630) filter set (including dichroic beamsplitter) | Chroma Technology | ~1500 |
| *openAF* autofocus components (using cage system) | Various | 5064 |
| Piezo Z-stage (PI Q-545.140) with controller (PI E-873.1AT) | Physik Instrumente | 3188 |
| Motorised x-y stage (Zaber ASR100B120B) with Controller (Zaber X-MCB2) | Zaber | 7660 |
| Transillumination mount and white LED with controller | Cairn Research Ltd | 1513 |
